# Supplementary material for: Associations of Single-Nucleotide Polymorphisms in Slovenian Patients with Acute Central Serous Chorioretinopathy
Source: Genes (Basel). 2021 Dec 25;13(1):55. doi: 10.3390/genes13010055 (PMC8774639; doi:10.3390/genes13010055)
Supplement: Supplementary file 1 [file genes-13-00055-s001.zip › genes-1513976-supplementary.pdf]

Supplement

Table S1. Oligonucleotide primer sequences for the High Resolution Melt (HRM) analysis

SNP	Primer sequence		Length (bp)
NR3C2 rs5522	Forward:	TTCAGAGGGCTGCAAACA	124
	Reverse:	ACACGCCCTTGAGATCATTTA	
CFH rs1329428	Forward:	AGTGGTCTAGATAGACTCTGGAT	112
	Reverse:	CACAAACTTGAGTGCCCTAAC	
CFH rs3753394	Forward:	ACAATAGACCCGAATAGAGTTTGA	119
	Reverse:	CTAGGGAAATTCTCCGTTGGAA	
CFH rs1065489	Forward:	TGATGTCATAGTAGCTCCTGTATTG	200
	Reverse:	ACTTCTTCTCCATACTGATAACTGTC	
CDH5 rs7499886	Forward:	AGCTGCTCTTATGTGAAATACCT	121
	Reverse:	ATCATGAGCCTCTGCATCTTC	
CFH rs800292	Forward:	AGGCACCCAGGCTATCTATAA	97
	Reverse:	TGGATTAAGAGCAACCCATTCT	
ARMS2 rs10490924	Forward:	GACAAGCAGAGGAGCAAACT	93
	Reverse:	AGGAGAGAAGAAGGCTGGTAA	
TNFRSF10A rs13278062	Forward:	CGGAATGAAGGACACAGAGG	135
	Reverse:	CAGAGCTTGACTCCATCTCAAA	
CFH rs1061170	Forward:	CCTTTGTTAGTAACTTTAGTTCGTCTTC	250
	Reverse:	GATGGCAGGCAACGTCTAT	
COL4A3 rs10178458	Forward:	AATAATAAGAAACTTTGTATGT	106
	Reverse:	GGGAATTAGGCATGCAAA	
COL4A3 rs55703767	Forward:	GACCCATTTCTTTTTGTTCT	110
	Reverse:	AAAATAGGCTATTAGGGAGA	
COL4A4 rs2229814	Forward:	TATAGAAGACAGTCAGAAAA	181
	Reverse:	TAGAAATTCTACCTTTGGTG	
COL4A4 rs2229813	Forward:	AAAGAAACTTATTAAGCCTT	153
	Reverse:	TTGTTATTTTATGCTGTTTA	

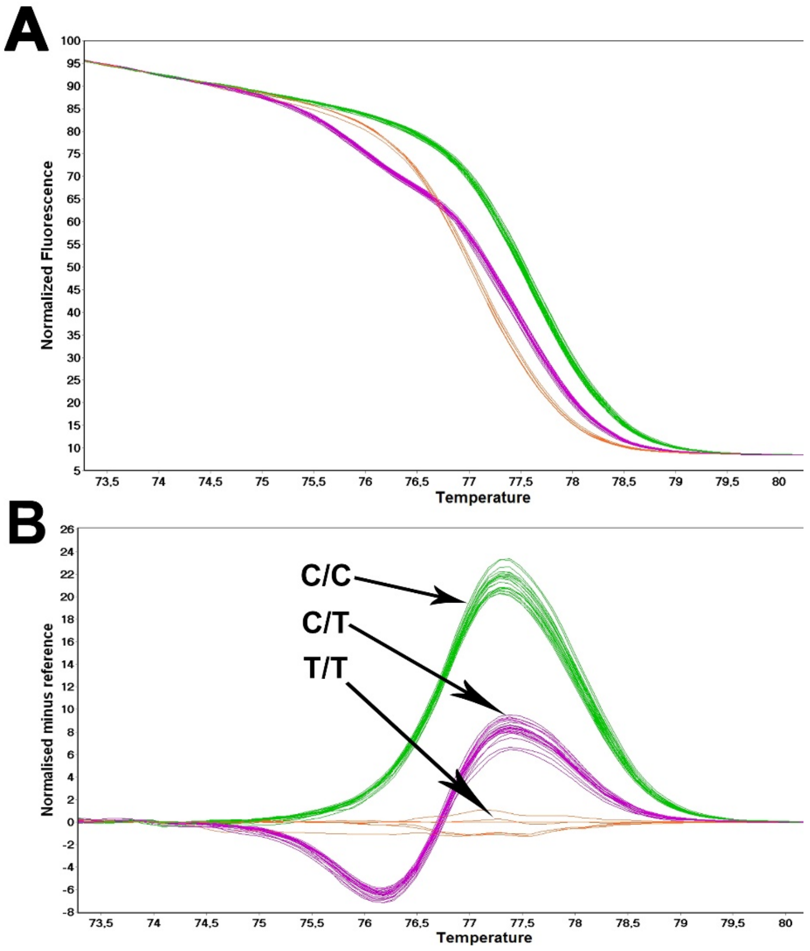

Figure S1. Example of the High Resolution Melt (HRM) analysis with distinct genotypes of CFH-rs1329428. Normalized high-resolution melting profile (A) together with differential plot (B).
